# Case of Traumatic Tetanus

**Published:** 1849-04

**Authors:** 


*Case of Traumatic Tetanus.* March 16th, 1848—Albert Fisher, German, aged 20 years, quite robust, proprietor of a "coffee house" in the 3d Municipality, entered ward 11, Charity Hospital. Says he ran a nail into the external portion of his right foot, near the junction of the "metatarsal" and first phalangeal bones of the 'little toe,' about five weeks ago; that the wound caused him considerable pain at the time, but soon healed without any difficulty; that, at the expiration of three weeks from the time of the reception of the injury, he was seized with violent tetanic spasms, together with slight pain in the original seat of injury; that on the following day a physician laid open the old cicatrix, and extracted a portion of the nail which had been imbedded there during all this time, that the spasms were now somewhat mitigated, but he was confined to his bed with constant rigidity of the back, jaws and hip joints, together with frequent lancinating pains in the lumbar region.

In this condition I find him this morning. He complains bitterly of insomnia, begs to have something which will induce sleep; every three or four hours he has an aggravation of the symptoms amounting to almost complete 'opisthotonos;' appetite good, bowels in good condition, perhaps a little torpid, skin perfectly natural, countenance wearing the distressed air so peculiar to these cases, complains much of the lancinating pain in his back, says they recur every three minutes.

*Treat.*—Ol. Ricin. f. ℥ i.—Tinct. Opii. gtt. xxx. every hour, after the free operation of the oil, until sleep is induced; low diet, Emplast. Vesicat to back.

17th. Says he feels better, but is drowsy and dull, oil operated freely, commenced taking laudanum at 12 M. yesterday, and repeated, as ordered, every hour until 12 at night, when he fell aleep and slept soundly until 6 this morning, pain in back not so intense, rigidity the same.

*Treat.* Moderate dose of magnesia and rhubarb, tinct. opii. gtts. lx., to be takent at 8 P. M., half diet.

18th. Not so well, slept very little last night, complains of the stupefying effects of the laudanum, pain in the back returned.

*Treat.* I now resolved to try the *internal* administration of chloroform, and ordered, accordingly, gtts. xxx to be given at once in one ounce of mucilage, and to be repeated every two hours, with gtts. x additional at each dose, until sleep is induced; blister, also, to be reapplied, it having taken but slight effect before.

19th. Took four doses of chloroform as ordered, and fell asleep about an hour after the first dose, slept soundly until 1 o'clock this morning, says he thinks he would have slept before, but for the irritation kept up by the blister; looks bright, feels much refreshed, back much relaxed, hip joints nearly entirely so, no unpleasant result from taking the chloroform.

*Treat.* Chloroform repeated as before, beginning with gtts. lx., half diet.

20th. Slept several hours yesterday, and soundly all night, feels much better, rigidity almost the same.

*Treat.* Chloroform gtts. c. at night, rhubarb and magnesia.

21st. Had several spasms yesterday before taking the chloroform, complains much of the pain consequent, countenance more distressed, had several spasms this morning, rigidity the same.

*Treat.* Chloroform gtts. c. to be taken at 12 M., and repeated at 4 and 8 P. M., if sleep is not induced.

22d. Took two doses of chloroform as directed, and slept from 4½ P. M., to 12 or 1 this morning; spasms quite frequent this morning, amounting to almost decided 'opisthotonos;' was aroused from his sleep by these spasms several times last night, much pain, is evidently growing worse.

*Treat.* Quin. sulph. grs. xxx.
Tinct. opii. gtts. lx.
Mucil. acac. f. ℥ i., M.
S. Take at 10 A. M.

5 P. M. Much better, but four or five spasms during the day, and these much mitigated, pain much relieved, countenance more cheerful, no 'tinnitus aurium,' or other disagreeable effect from the use of quinine.

*Treat.* Repeated the quinine, &c.

23d Very much improved, had but one or two slight spasms since I last saw him, scarcely any pain, slept well last night, is delighted with the remedy.

*Treat.* Same dose as before, to be taken at 10 A. M., and 3 and 9 P. M.

5 P. M. Has taken two doses as ordered, feels better every hour, spasms scarcely perceptible, no pain, has slept well during the day, no unpleasant symptom from quinine, feels 'a little drunk.'

24th. Still improving, rigidity of back very slight, no pain, slept well all night, looks cheerful. *Treat.*—Same dose quinine at 12 M. and 8 P. M.

25th. Marked improvement, no more spasms, no pain, rigidity very slight, slept well, looks bright, says he is nearly well.

Treatment same as before.

26th. Patient is walking about the ward, improving rapidly. Same treatment continued.

27th. Suspended the quinine, and ordered good diet, small dose of oil.

30th. Discharged him, *well*

*Observations.*—This is the first instance, I believe, wherein chloroform has been *internally* administered in the City of New Orleans, and notwithstanding its failure to effect the desired end, it may not be uninteresting to the profession to know the results of its exhibition. It evidently possesses the power of producing anesthesia when thus administered, and my limited observation leads me to believe that the process of inhalation may ere long be superceded by the method under consideration. The patient experienced no disagreeable effect whatever from its use; there were none of the distressing symptoms which I have so often seen consequent on the inhalation of the agent, viz., wild delirium, convulsive action of the muscles of the extremities, and astonishingly increased activity of the circulation. The effect appeared to be more gradual, the patient passing off into a sweet and refreshing sleep without being subjected to previous excitement.

The dose administered was much larger than in any previous instance that I know of, and, I think, may have been considerably increased without incurring any risk of unpleasant consequence.

In relation to the administration of quinine in this case, I cannot refrain from calling the particular attention of the skeptical reader to a few facts extracted from the above 'Notes.'

1st. The patient, at the time of the adoption of the quinine treatment, was evidently growing worse. 2d. That marked improvement followed the first dose. 3d. That, so far from any disagreeable symptoms having followed said dose, the patient actually expressed himself as 'happy' whilst under its influence, and begged to be kept constantly in this condition. 4th. That an hour and a half after having taken two thirty grain doses, at an interval of five hours, he said he felt even better than before, no other disagreeable sensation than that of being 'a little drunk.' 5th. That on the morning after having taken ninety grains within ten hours he expressed himself as much improved in every way, and *was* so. 6th. That after this he took sixty grains daily, in two doses, for three days, without experiencing a single one of the much dreaded effects of the medicine, and was not only relieved of the tetanic symptoms, but improved most wonderfully in appearance, slept well, had a fine appetite, and digested the strongest food with ease.—*New Orleans Med. and Surg. Jour.*

